# Identifying core mental health services required for adult justice-involved populations in Australia: a Delphi study

**DOI:** 10.1080/13218719.2024.2330049

**Published:** 2024-05-07

**Authors:** Charlotte Comben, Zoe Rutherford, Carla Meurk, Julie John, Kevin Fjeldsoe, Kate Gossip, Sandra Diminic

**Affiliations:** a School of Public Health, The University of Queensland, Brisbane, QLD, Australia; b Mental Health Services Research Group, Queensland Centre for Mental Health Research, Brisbane, QLD, Australia

**Keywords:** Delphi, forensic mental health, forensic mental health services, health service planning, prison mental health services

## Abstract

This study aimed to define required forensic mental health service types in Australia. A staged Delphi process was used, including: (1) reference group consultation to establish a set of core forensic mental health service types; (2) Delphi survey to seek consensus on identified service types and definitions among forensic mental health stakeholders in Australia; and (3) amendment of service definitions in response to survey feedback. Nine forensic mental health service types were identified. Consensus (>80% agreement) was achieved among Delphi respondents that all services were core components of an ideal forensic mental health system. These included forensic bed-based (acute, sub-acute, non-acute, non-acute community), forensic community, outreach, prison and court liaison services. The high levels of agreement on service types are notable considering legislative differences impacting on design and operation of services across jurisdictions. The findings could contribute to the evidence base required for needs-based planning of forensic mental health services.

## Introduction

Adults with mental illness are over-represented within the criminal justice system, presenting significant health and welfare concerns (Butler et al., 2006; Fazel & Seewald, 2012; Stewart et al., 2021). Justice-involved populations often have other health or complex social needs (Baranyi et al., 2018; Binswanger et al., 2009; Fazel et al., 2006; Productivity Commission, 2021). Furthermore, incarceration is associated with negative health outcomes, including increased risk of suicide and mortality following release from prison (Fazel et al., 2016; Forsyth et al., 2018). In Australia, First Nations people are overrepresented in the criminal justice system, particularly in prisons (Australian Bureau of Statistics, 2024), and are known to have disproportionally higher mental health needs (Heffernan et al., 2012).

Despite high levels of needs, justice-involved individuals experience limited access to the health care available to other populations (Kinner et al., 2012; Linnane et al., 2023). In Australia, this limited access results in part from the exclusion of people in custody from Medicare, the country’s universal health care program, as well as underinvestment in services and workforce (Davidson et al., 2020; Plueckhahn et al., 2015). Individuals released from prison utilise health services at higher rates than the general population (Carroll et al., 2017; Snow et al., 2022). The provision of appropriate, comprehensive forensic mental health care is essential for preventing poor health outcomes and further criminalisation (Crocker et al., 2017; Munetz & Griffin, 2006).

Forensic mental health services provide mental health care to people engaged with (or at risk of engaging with) the criminal justice system who have mental illness (Every-Palmer et al., 2014). This includes mental health care in prisons, inpatient and community specialist forensic mental health care, and court liaison services. The care provided is dependent on an individual’s severity of mental illness, security requirements, stage of engagement with the criminal justice system, and available resources within the mental health system (O’Donahoo & Simmonds, 2016).

Forensic mental health service models vary by jurisdiction (Every-Palmer et al., 2014; Salize et al., 2023; Salize & Dreßing, 2005; Sampson et al., 2016), largely due to the unique role of policy and legal frameworks in defining and engaging with justice-involved populations, and different ways in which boundaries are drawn between forensic and general mental health populations and services (Jansman-Hart et al., 2011; Khan et al., 2018). Globally, there is no guideline for the ideal design of forensic services or systems (Crocker et al., 2017; Dean, 2020). The lack of availability of unified definitions to describe service models in a clear and consistent manner has been identified as a knowledge gap (McKenna & Sweetman, 2020).

Within Australia, state and territory forensic mental health systems share common elements across models of care (Dean, 2020), but differ in precise models of service delivery due to varying legal frameworks, geography and demographics (Bartels et al., 2018; Ellis, 2020; Hanley & Ross, 2013). For example, some jurisdictions employ dedicated mental health teams within prisons, while others use in-reach into prisons by specialist forensic mental health services (Every-Palmer et al., 2014). Community forensic mental health services also vary; some jurisdictions provide direct specialist case management under a ‘parallel’ model of care (i.e. parallel to general mental health services), whereas others operate under an ‘integrated’ model of care, in which forensic teams provide specialist consultation liaison in-reach to general mental health services for the care of forensic consumers (Ellis, 2020; Latham & Williams, 2020). Service models are necessarily varied, due to the country’s unique geography and variable population density. These factors greatly affect health service access and delivery and necessitate innovative models of service delivery, such as the hub-and-spoke model (Australian Institute of Health and Welfare, 2023; Elrod & Fortenberry, 2017). Considered together, differences across jurisdictions pose challenges for coordinated planning of services.

A national model to plan forensic mental health services in Australia is needed to systematically estimate required resources to meet population needs (Whiteford et al., 2023), ensuring that services are available to those who require them (Productivity Commission, 2021). Coordinated service planning is also important to bring forensic mental health services to parity with general mental health services, which have common guidelines and planning requirements. A national model for forensic mental health service planning that is based on commonalities across jurisdictions would support the use of clear and consistent terminology among health planners. Such a model would allow the establishment of standardised national benchmarks to improve care and enable comparison across jurisdictions. One example of a needs-based planning model is Australia’s National Mental Health Service Planning Framework, which is used to support planning to ensure adequate health service resourcing is available to meet population needs (Diminic et al., 2022). In recent years there have been calls to develop modelling for justice-involved populations, which was not included in initial versions of this model (David McGrath Consulting, 2019; Davidson et al., 2020; Productivity Commission, 2020; Southalan et al., 2020).

To achieve needs-based planning in Australia, an understanding of the core forensic mental health service types that should be available, and definitions of these service types, is required. National principles to guide the provision of forensic mental health care discuss generally the types of care that should be provided; however, specific service types are not included (Australian Ministers’ Advisory Council Mental Health Standing Committee, 2006). Furthermore, a recent consultation found many forensic mental health stakeholders were unaware of these principles (Queensland Forensic Mental Health Service & Queensland Centre for Mental Health Research, 2022).

## Aims

This study aimed to establish and define the core service types that should exist within an ideal forensic mental health system and seek consensus among a range of participants with experience in forensic mental health services. This information could contribute to the evidence base required to enable needs-based planning of forensic mental health services.

## Method

### Study design

A Delphi process including expert consultation and an online survey was used (Msibi et al., 2018; Thomas et al., 2014; Westby et al., 2013; Young et al., 2022). The Delphi process was chosen as it allows a wide range of geographically diverse participants to voice anonymous opinions with the aim of generating consensus on items of interest to the research question (Barrett & Heale, 2020; Iqbal & Pipon-Young, 2009; Jünger et al., 2017; Waggoner et al., 2016). Services responding solely to the substance abuse or intellectual disability needs of people within the criminal justice system were considered out of scope. Ethics approval was obtained through the University of Queensland (Ethics IDs: 2020/HE001887 & 2022/HE002250).

### Stage 1: Reference group consultation

Initial consultation was conducted through a broader project to scope the feasibility of developing a needs-based planning model for forensic mental health services in Australia. A panel of senior forensic mental health clinicians were identified through their leadership roles within forensic mental health services and invited to participate via email in a series of three one-day meetings during 2018. Additional delegates were nominated by invitees to attend meetings due to their specialist knowledge where relevant, including forensic mental health service clinical and operational directors. During meetings, reference group members were asked to draw on professional experiences and, through iterative group discussion, generate a set of provisional inputs for a forensic mental health service planning model. At the conclusion of the three meetings, a set of provisional modelling inputs were identified, including nine core forensic mental health service types with accompanying service definitions. Results from this consultation were used in the development and implementation of a Delphi process.

### Stage 2: Online survey

The survey was conducted over three rounds between November 2020 and January 2021 to seek agreement on the service types and definitions developed in Stage 1.

#### Recruitment

Purposive and snowball sampling were used to identify prospective participants with expertise in forensic mental health care. A reference group, consisting of members of a network of senior clinical leaders in forensic mental health, identified key stakeholders to invite from forensic mental health service settings within each Australian jurisdiction. Stakeholders from forensic mental health services, non-government organisations, judicial services, corrective and custodial health services, and police were invited, as well as people with lived experience within, or supporting someone within, the forensic mental health system. Participants from the Stage 1 reference group consultation were also invited. Invitees were asked to circulate the invitation within their networks.

#### Survey design

The survey questionnaire was developed by the research team and delivered via Checkbox online survey platform. Survey content is summarised in Table 1. The survey was structured according to key settings in which forensic mental health services are provided (i.e. bed-based, community, prison, court); participants were able to select survey sections to complete based on their expertise. Briefing documents explaining key concepts and definitions were developed to assist participant understanding and interpretation of questions.

**Table 1.** Example Delphi survey questions from each round.

Survey round	Example questions
1	Is this service type a core component of a comprehensive forensic mental health system? *(Yes/No)* Are all key functions of this service type described above? *(Yes/No)* Please provide any comments related to the key functions of the service type. *(Free text)* Are there any services not included in this model you believe to be a core service within a comprehensive and ideal forensic mental health service system? *(Yes/No)* Please describe any service types missing from this model. *(Free text)*
2	Please rate the extent to which you agree that each of the following statements is a key detail within the service attributes. *(Four-point Likert scale)* In Round 1, respondents suggested the following additional service types as necessary for inclusion within a comprehensive forensic mental health service model. Please rate your agreement on the inclusion of the following service types. *(Four-point Likert scale)*
3	Please rate the extent to which you agree that each of the following statements is a key detail within the service attributes. *(Four-point Likert scale)* In Round 2, your agreement was measured on the additional service types suggested by respondents in Round 1. The additional service types you agreed were necessary for inclusion within a comprehensive forensic mental health service model are listed below. Please rank these service types based on the importance of their inclusion within a comprehensive forensic mental health service model. *(Five-point Likert scale)*

#### Survey process

Each survey round was open for up to three weeks. The first round was sent to all invitees; second and third round questionnaires were sent to all participants who had completed the previous round, so results were not confounded by the addition of new respondents who did not have the context from prior rounds. Between survey rounds, qualitative and quantitative responses were analysed to highlight areas of agreement and disagreement regarding service types and definitions. Qualitative responses were synthesised, and key themes were refined to create new consensus items (i.e. amendments to service definitions or additional service types), which were presented back to participants in subsequent rounds. The Round 2 and 3 questionnaires used four-point Likert scales (‘strongly disagree’, ‘disagree’, ‘agree’, ‘strongly agree’) to seek consensus on newly proposed items. Round 3 also included one question with a five-point Likert scale to rank additional core service types.

#### Analysis

There is no uniform definition for consensus in Delphi studies (Barrett & Heale, 2020; Drumm et al., 2022; Niederberger & Spranger, 2020). Here, consensus was defined as at least 80% of respondents answering the same way (i.e. ‘yes’ or ‘no’ in Round 1; agree/strongly agree or disagree/strongly disagree in Rounds 2 and 3), as used in previous studies (Penm et al., 2019; Thomas et al., 2014; Watson et al., 2019). Descriptive statistics were generated for Likert scale response questions (mean, standard deviation and range). All items that did not achieve consensus were presented back to participants in subsequent rounds with the level of agreement reached for their re-scoring. Items that did not reach consensus after the third round were discussed with a reference group during Stage 3.

### Stage 3: Reference group consultation

Two video-conference meetings were convened with a reference group of senior forensic mental health clinicians to discuss survey results. Membership of this reference group was drawn from the network who guided the recruitment of participants during Stage 2. Some, but not all, participants in this reference group took part in the Stage 1 consultation. During meetings, the consensus results for all items (e.g. service types, edits to service definitions, and suggested additional service types) were reviewed. The reference group made judgements as to the appropriateness (i.e. correctness and distinctiveness) of each item based on their professional experience. Items deemed to be inappropriate were not included in the final set of core service types and descriptions. This consultation added additional insight and context for survey responses, including discussing possible rationales for why some items did not achieve consensus.

## Results

**Figure 1.**
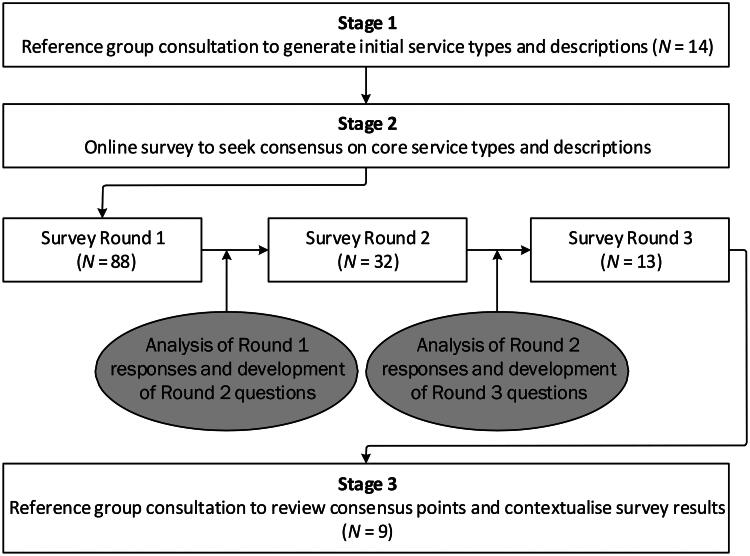
Study process and participation.

The Stage 1 reference group consultation included 14 participants from seven out of eight Australian jurisdictions who all held senior positions within forensic mental health services. The Stage 2 online survey included participants from all Australian jurisdictions. Over 200 participants accessed the survey; however, respondent numbers decreased with each round (Figure 1). Across all survey rounds, participants from forensic mental health services were the largest group. Participants also included police, magistrates and a prison director. There was lived experience representation only in Round 1 of the survey (5% of participants identified as a consumer or carer). The Stage 3 reference group consultation included nine participants from five jurisdictions who all held senior positions within forensic mental health services.

### Core service types

All nine forensic mental health service types developed through the Stage 1 reference group consultation achieved consensus during Round 1 of the survey (Table 2). The number of respondents to questions about each service type varied, ranging from 9 to 41 (Table 2). The largest number of participants answered questions about the Community Forensic Outreach Service (*n* = 41); the fewest answered questions about non-acute bed-based service types (*n* = 9). The only service type that all participants agreed was a core service type was the Court Liaison Service. The Prison Mental Health Service had the lowest level of agreement among repondents (88%). However, two of the three respondents who did not agree this was a core service type provided qualitative feedback suggesting they did think it was a core service type, but disagreed with the service definition. Qualitative responses to this effect included: ‘*a lot more detail is required to make functional models of care in prisons*’ and ‘*while the service attributes aren’t wrong, they’re inadequate to describe the level of care*.’ There were three instances where this apparent misalignment within individual responses occurred for other core service types.

**Table 2.** Agreement on core service types for forensic mental health services during survey Round 1.

Service type	Respondents^a^ (*n* = 88) (*n*)	Core service agreement^b^ (%)	Service definition agreement^c^ (%)
Court Liaison Service	37	100	81
Community Forensic Outreach Service	41	98	63
Community Forensic Mental Health Service	35	97	83
Acute Forensic Management Unit	27	96	91
Acute Forensic Assessment Unit	23	93	81
Sub-Acute Forensic Unit	11	91	82
Non-Acute Forensic Unit	9	89	67
Non-Acute Forensic Rehabilitation Unit	9	89	78
Prison Mental Health Service	26	88	58

^a^Respondents were able to select the service types they answered questions about based on their expertise.

^b^Proportion of respondents who selected ‘Yes’ when asked whether the presented service type was a core component of an ideal forensic mental health system.

^c^Proportion of respondents who selected ‘Yes’ when asked whether all key functions of the service were included in the service definition.

^d^For full definitions of service types see Appendix A.

### Definitions of core service types

Agreement about whether all key functions were described for each service type highlighted heterogeneity among survey participants (Table 2). In Round 1 of the survey, the highest level of agreement among respondents was for the preliminary definition of the Acute Forensic Management Unit (91%); the lowest was for the Prison Mental Health Service (58%). Qualitative feedback suggesting amendments to service definitions was at times contradictory: for example, opposing feedback was received about the appropriateness of the Prison Mental Health Service model described (Box 1).

Box 1 Examples of heterogeneity in survey qualitative feedback on proposed service definitions

**Table:** 

Referring to Community Forensic Outreach Services: Respondent 1: [It is] *‘essential that forensic specialist knowledge is available to community services*.*’* Respondent 2: [Services should provide] *‘education for stakeholders* *–* *Police, ambulance, court, GP etc*.*’* Respondent 3: *‘I disagree with providing specialised training to general mental health service and agencies in the criminal justice system.’* Referring to Prison Mental Health Services: Respondent 4: *‘wouldn’t it be great to have a functioning service that provided those services and in reality interfaced with the public community teams in the metro and country regions*.*’* Respondent 5: *‘This model of care is contentious as it does not outline what a prison mental health unit entails. It is against current guidelines*.*’*

Table 3 demonstrates the translation of qualitative feedback into new consensus items (i.e. attributes to include in service definitions), the level of consensus reached among survey participants, and the feedback provided by the Stage 3 reference group. Commonly received qualitative feedback was translated into new service attributes; however, not all new service attributes achieved consensus during subsequent survey rounds. For example, Round 1 Delphi respondents suggested that Court Liaison Services should provide assessments to determine fitness for trial and cognitive status. However, when this service attribute was proposed in survey Round 2, only 55% of respondents agreed it was a core service attribute (Table 3).

**Table 3.** Example qualitative feedback from Delphi consultations on proposed forensic mental health service definitions.

Service type	Summary definition of service (from Stage 1 reference group meetings)	Feedback received (during survey Round 1)	New service attribute proposed (during survey Round 2)	Consensus reached in survey (agreement)?^	Result of Stage 3 reference group consultation
Court Liaison Service	This service aims to provide mental health advice, assessments, diversion and referral for people who have been charged with an offense. Key attributes include: Delivered in lower courts, police custody and prison settings Services provide or facilitate specialised advice to the court regarding the impact of a person’s mental health or intellectual capacity on their offending behaviour and ability to take part in legal proceedings	*‘* *specialist psychological assessments for fitness and cognitive status* *’* *‘* *Provision of mental state assessments is important in the diversion program. Ensuring defendants receive treatment asap.* *’*	Court Liaison Services should be responsible for providing specialist psychological assessments to determine fitness and cognitive status.	No (55%)	Do not include as key attribute Reference group advised this attribute fits better within the function of the Community Forensic Mental Health Service.
Community Forensic Outreach Service	This service aims to provide clinical assessment, structured risk assessment, liaison, support, advice and risk management planning support for people who are in the care of mainstream mental health services or in contact with other agencies and who are engaging in (or at risk of engaging in) risky behaviours. Key attributes include: Services are delivered to mainstream mental health services and/or other referring stakeholders at the interface of mental health and the criminal justice system This service is typically a function of Community Forensic Mental Health Services	*‘* *case management of high risk clients should be an integral part of the model of care* *’* *‘* *manage a case load of those patients identified as high risk and beyond the expertise of mainstream services* *’* *‘* *Dedicated case management of a small number of high risk offenders […] on specific forensic orders may not be suitable for mainstream community [mental health services].* *’*	Community Forensic Outreach Services case manage a cohort of high-risk patients whose needs are beyond the expertise of mainstream mental health services.	No (75%)	Do not include as key attribute Reference group advised this statement does not reflect the intention of this core service, which was designed with a consultation liaison function. These services provide assessment and specialist advice to mainstream services. Case management is provided by Community Forensic Mental Health Services.
Acute Forensic Assessment Unit	This service aims to provide short to medium-term inpatient assessment and treatment planning services for people in custody who are experiencing severe episodes of mental illness complicated by alleged or proven offending. Key attributes include: Clinical focus on decreasing acuity to a level that the individual can be treated in another environment (such as outpatient care by prison mental health services) Provided in a setting which balances consumer welfare and community safety	*‘* *Could have more of a trauma aware focus* *’* *‘* *Trauma informed practice needs to be specified within the service attributes.* *’*	The Acute Forensic Assessment Unit provides trauma-informed practice.	Yes (100%)	Include as key attribute Reference group advised this attribute should be included in all bed-based service definitions.
Non Acute Forensic Unit	This service aims to provide long term treatment and rehabilitation in a safe, structured environment for individuals with severe mental illness associated with significant violent or other serious offending behaviour. Key attributes include: Located within high security forensic hospitals Programs build and test an individual’s ability to manage their treatment needs as well as relationships, occupational capacity and activities of daily living within the safety of a secure perimeter Average length of stay would be at least 12 months (may be impacted by legal circumstances depending on the jurisdiction)	*‘* *Dislike the model suggesting it has to be a prolonged stay (i.e.* *12 months +)* *’*	The average length of stay within a Non Acute Forensic Unit is dependent on legal circumstances and jurisdictional differences, but would generally be at least 12 months.	Yes (86%)	Do not include as key attribute Reference group advised this attribute should not be included as it was very similar to a statement in the originally drafted service definition and the phrasing of the first statement was preferred.
Non-Acute Forensic Rehabilitation Unit	This service aims to provide long term rehabilitation and treatment for people with severe mental illness associated with significant violent or other serious offending behaviour. Key attributes include: Located in a low secure environment and in a setting that faciliates access to clinical support services Offers rehabilitaiton programs aimed at maximising individual functioning and minimising likelihood of repeat ofending related to recurrent mental illness	*‘* *There is no mention of the role of NGO support in these facilities and the model of interactions with health and these services in this setting.* *’*	Support provided by Non-Government Organisations (NGOs) is a key aspect of Non Acute Forensic Community Rehabilitation Units.	Yes (88%)	Include as key attribute
Prison Mental Health Service	This service aims to provide mental health services to people in prison. Key attributes include: Facilitates access to mental health care in general prison environments Provides reception screening for presence of mental illness; early identification of mental illness; assessment, treatment and care; and transfer of consumers to hospital if necessary	*‘* *7 days per week and extended hours required* *’* *‘* *prison MH can be responsible for crisis assessment for prisoners at risk of suicide and self harm. So need to be available extended hours and weekends to work with Corrections to put in safety planning* *’*	Prison Mental Health Services should be available 7 days per week, operating on an extended hours schedule.	No (67%)	Include as key attribute with minor amendment: Edit text to specify workforce can be ‘on site or on call.’

^Proportion of respondents who agreed or strongly agreed that the new service attribute should be included in the service definition.

Not all new service attributes that achieved consensus during the survey were considered appropriate to include in the final service definition by the Stage 3 reference group. Survey participants suggested 50 new service attributes across all service types; 33 achieved consensus among survey respondents; 30 were deemed appropriate for inclusion in service definitions; 6 service attributes that did not achieve consensus among survey respondents were deemed appropriate for inclusion in service definitions with minor amendment by the Stage 3 reference group.

### Additional core service types

Of Round 1 survey participants, 44% indicated that there were additional core service types not included. There were 10 additional service types suggested, however, only one reached consensus (i.e. ⩾80% agreed or strongly agreed it was a core service type) in subsequent survey rounds (Forensic Step Down (Sub Acute) Unit). When additional suggested service types were reviewed by the Stage 3 reference group, none were deemed appropriate to include as core service types. The reference group suggested that the function of the Forensic Step Down (Sub Acute) Unit was already included within existing bed-based service types. Additional service types suggested that did not reach consensus included: court report writing services, which the reference group suggested are included within the function of Court Liaison Services; specialist psychological intervention services, which the reference group suggested are included within the function of the Community Forensic Outreach Service and the Community Forensic Mental Health Service; and police co-response services, which the reference group suggested are not a core function of forensic mental health services.

### Final service descriptions

Final core service types and definitions are included in Appendix A. In each service description, italics indicate text that was added or amended through the Delphi and/or Stage 3 reference group consultation process. These final service descriptions represent agreed core forensic mental health services that should be available in Australia, despite jurisdictional differences that may exist in current service models or operational factors.

## Discussion

Nine core service types were identified across forensic mental health settings in Australia, with a high level of agreement. Bed-based services include Acute Forensic Assessment, Acute Forensic Management, Sub Acute Forensic, Non Acute Forensic and Non Acute Forensic Community Rehabilitation Units. Community-based services included Community Forensic Mental Health and Community Forensic Outreach Services. The Prison Mental Health Service and Court Liaison Service were also identified as core services. All service types reached consensus among participants, and heterogeneity in responses about service definitions was addressed through reference group consultation. The identified service types broadly align with Australian forensic mental health services described in previous literature (Davidson et al., 2016; 2020; Dean, 2020; Ellis, 2020; Every-Palmer et al., 2014; Productivity Commission, 2020). They also align with the continuum of ‘balanced’ forensic mental health care outlined by Crocker et al. (2017).

Bed-based services are labelled by acuity and distinguished by level of security, as is done in Australia, New Zealand, England, Wales, Scotland and Ireland (Crocker et al., 2017; McKenna & Sweetman, 2020). This has been referred to in the literature as the ‘standard model’ of bed-based forensic mental health care (Kennedy, 2022). However, beyond a focus on procedural, relational and environmental security, there is little known about models of care for bed-based services in Australia (Khan et al., 2018). The services described here target the dual responsibility of forensic mental health services to balance risk while supporting individual recovery in terms of both mental health and criminogenic needs (i.e. personal factors related to an individual’s offending behaviour, such as antisocial behaviour).

The Prison Mental Health Service aligns with the essential requirements of service provision in prison settings, including screening, triage, assessment, intervention and re-integration, as outlined by Forrester et al. (2018) and the Prison Model of Care in northern New Zealand (Pillai et al., 2016). Although the provision of mental health care in prisons is an essential component of forensic mental health care globally (World Health Organisation, 2008), the definition of a Prison Mental Health Service included in Round 1 of the survey achieved a low level of consensus. The prison mental health workforce in Australia is known to be underresourced (Davidson et al., 2020). It is possible that Delphi participants referred to their own experiences of working in prisons and found the service definition to contain functions or aspects of care that are not currently provided in their jurisdiction due to resourcing constraints. Prison health services should be aligned to the structure of health services in the community, i.e. primary health care, secondary health care and specialist, tertiary care (Levy, 2005). Collaboration with general prison health services to deliver primary care functions is an essential component of comprehensive prison mental health care.

The Court Liaison Service was also identified as a core service type, perhaps due to its importance in diverting people out of the criminal justice system wherever possible and offering onward referral to appropriate mental health services. The service definition is aligned with service aims and models both in Australia and internationally (Chaplin et al., 2022; Davidson et al., 2016). Round 1 survey participants agreed that all key functions were described in the service definition, suggesting that the service definition highlighted commonalities in service provision across Australia. Most additional attributes suggested by participants in Round 1 to enhance the description of this service did not reach consensus in Rounds 2 or 3. This is possibly because the suggested service attributes were highly specialised and not common across all jurisdictions.

The delivery of Community Forensic Mental Health Services in Australia differs across states and territories (Brett et al., 2007; Ellis, 2020). Despite these differences, consensus was achieved on the service definition provided in survey Round 1, highlighting that participants were able to agree on the key attributes of this service type. The definition of this service type was drafted to reflect the service function while remaining agnostic to the various models of service delivery (i.e. parallel vs integrated). The description of this core service type is aligned with that for England and Wales (Latham & Williams, 2020).

Although they serve distinct functions, Community Forensic Outreach Services (CFOS) are related to Community Forensic Mental Health Services. CFOS may be a function of, or specialist team within, Community Forensic Mental Health Services and provide clinical assessment, structured risk assessment, liaison, support, advice, and risk management planning support for consumers who are in the care of mainstream mental health services or in contact with other agencies and who may be at risk of offending. The differing service delivery arrangements across jurisdictions may have resulted in confusion among Delphi participants. In the first round of the Delphi, a high level of agreement was observed for the Community Forensic Outreach Service being a core service type; however, the level of agreement for the service definition was among the lowest. This was further evidenced by the qualitative feedback that CFOS services provide a case management function. However, reference group contextualisation established that case management is not a core function of CFOS, because it is separately described under the attributes of Community Forensic Mental Health Services. This discrepancy may result from the influence of participants’ local experiences on their interpretation of the core service types.

A strength of this study was the diversity of participants consulted, including representation from all Australian jurisdictions. The heterogeneity of responses about service definitions highlights challenges in undertaking consensus work; the included Stage 3 reference group consultation was therefore critical for aligning feedback and revising definitions with best practice principles.

Several limitations were identified. First, there was limited lived experience involvement in this research. The sampling method limited the recruitment and engagement of lived experience stakeholders. Although invitations to participate in the survey were disseminated to lived experience organisations and peak bodies, the invitation may not have been shared through their networks, further limiting the opportunity for lived experience participation. This limitation is important to note, as lived experience stakeholders’ perspectives may be different from those of the stakeholders who participated in this study.

Second, there were small numbers of participants and a drop in survey response rates across the three rounds (coinciding with the end-of-year holiday period). This included loss of the small number of lived experience participants. However, the small number of participants in this study is not surprising, given the specialist topic: not all forensic mental health stakeholders would have had interest or expertise to participate in the survey or would have received the invitation. Furthermore, the potential participant pool for this study was small, given that there are known workforce shortages in Australia’s mental health sector (Department of Health and Aged Care, 2022b), especially for forensic psychiatrists (Acil Allen, 2020).

Third, specific First Nations cultural expertise and consultation were not included in the reference groups or design of this study. Culturally safe and appropriate approaches to mental health care for First Nations people are essential (Page et al., 2022; Perdacher et al., 2019), requiring co-design and appropriate cultural consultation. A decision was made at the outset of the study to conduct separate consultations for First Nations people, including appropriate cultural expertise at their core. The study described here takes a generalist approach that will be supported by additional cultural consultation and does not reflect the steps required to establish core forensic mental health service types for First Nations people.

Fourth, some intersectional service types were not identified during this process, such as services supporting the transition of justice-involved individuals back into the community and psychosocial support services provided by the non-government sector.

## Conclusion

This study has generated evidence that could be used to undertake needs-based planning of forensic mental health services in Australia by establishing a set of required service types and definitions, as has been called for in recent policies and reports (Department of Health and Aged Care, 2022a; Productivity Commission, 2020). This information could serve as a key input into a needs-based planning model for forensic mental health services in Australia.

A challenge of needs-based planning is the requirement of common definitions for core service functions. Attempting to generate consensus among participants from different jurisdictions who operate under different service working definitions posed a challenge. Service definitions were developed so their intent and function could be adapted to jurisdictional differences (i.e. defining the function of a service type and, to the extent possible, not specifying the specific setting or other operational factors).

Although undertaking needs-based planning of forensic mental health services in Australia will assist service planners to identify the services required, it is up to health services to properly resource and deliver services to appropriate cohorts. The core service types generated by this study provide health service planners with an understanding of the forensic mental health service types that should be available in their jurisdiction to justice-involved people. By comparing services currently available with the core service types, health planners can identify if there are gaps in their service offerings. The detailed definitions will support planners to accurately map their catchment’s services against the attributes of the identified core service types, to ensure they are comparing like-for-like, based on core functions rather than local service names or groupings. Implementation will then need to be further tailored to specific local legislative requirements and consider the efficient location, staffing and grouping of core service types for the size and distribution of local justice-involved populations.

However, the presence of differences does not preclude the development or use of a nationally consistent model. Although there are differences in forensic mental health service models across Australia, health planners need a consistent and common terminology. This consistency also helps to support the integration between forensic and general mental health services, for which a comprehensive national set of service descriptions exists (Comben et al., 2022). Internationally, the identified service types and definitions could be used to undertake similar needs-based planning activities. Establishing core service types and definitions helps to standardise functions and allows greater comparison across services internationally. It could also be useful in future research evaluating service model effectiveness and understanding outcomes of the identified core service types. Further work is needed to understand how the identified service types and definitions could be tailored to best meet the needs of First Nations people in the criminal justice system in Australia in a culturally safe and appropriate way. To support needs-based planning of forensic mental health services, this work should now be extended to identify the number of people in Australia likely to require each service type in any given year.

## Appendix A.

### Final service definitions

*Text* *set in italics* *was added or amended during the Stage* *2* *and* *3* *consultations.*

**1.** Community Forensic Outreach Service

Service attribute	Details
Services provided	Aims to provide services for a cohort of patients who have a mental illness, appear to have a mental illness or are at risk of developing a mental illness and are engaging in or at risk of engaging in behaviours that pose a serious risk to others. *Provides trauma-informed and culturally secure responses for a cohort who often face stigma and discrimination within mainstream mental health services.* The service provides clinical assessment, structured risk assessment, liaison, support, advice and risk management planning support for consumers who are in the care of mainstream mental health services or in contact with other agencies. Services are delivered to mainstream mental health services (bed-based and ambulatory) and may include delivery to other referring stakeholders at the interface of mental health and the criminal justice system. Includes provision of specialised training *and/or education* to mainstream mental health services *or other stakeholders (e.g. police, courts).* *May include sub-specialist programs, such as interventions for problem behaviours (i.e. personality disorders, paraphilias, sexual, stalking, fire setting) and fixated threat assessment.*
Key features	Services are provided by multidisciplinary teams and are usually provided as a state-wide service. Teams are a function of, or a specialist team within, a Community Forensic Mental Health Service. Teams have particular specialist expertise in the assessment and management of serious risk of harm to others associated with mental health problems. Caseload is determined by referral process and based on risk. *At the time of referral to the service, consumers should have an active diagnosis and be case managed by mainstream mental health services or other providers.* *Services may provide joint management of consumers with mainstream mental health services to aid in transition between services.*
Hours of operation	Business hours with after hours on-call emergency service provided by local mainstream or specialist forensic mental health services.
Population profile	Consumers often have a complex presentation of severe mental illness, with high levels of comorbidity particularly substance use disorders, severe personality disorders, cognitive impairment, and who pose a significant risk of harm to others.
Example service	Community Forensic Outreach Service (Qld)

**2.** Court Liaison Service

Service attribute	Details
Services provided	Aims to provide mental health advice, assessments, referral and diversion for people who have been charged with an offence. These services intervene early in the criminal justice process at the post-arrest and pre-sentence stages. *Court Liaison Services are responsible for seeking out individuals already within the court system who may require services. This may entail reviewing court lists for individuals who are already known to mental health services and/or attending court and making the service known to consumers and families.* *Court Liaison Services should provide services to higher-level courts (including Supreme Court), children’s courts, tribunals and drug and alcohol courts* and to lower courts (Magistrates’ Courts & Local Courts), police custody and occasionally in prison settings. Court liaison services: ○Conduct mental health assessments and may intervene to link individuals to mental health service providers. ○Provide or facilitate specialised advice to the court regarding the impact of a person’s mental health or intellectual capacity on their offending behaviour and ability to take part in legal proceedings. ○Provide advice to individuals before the court, their relatives/carers, service providers or legal representatives about issues related to mental health and relevant legislation. *Some services may include police custody support.* *Services may include oversight of therapeutic justice.*
Key features	Court liaison services are provided by multidisciplinary teams. These services operate at the interface between the lower courts, criminal justice system and general mental health services and have particular expertise in the interface between these systems and relevant legislation. Some services operate as part of the local area or district mental health service and others, particularly those serving large courts and watch houses will be a dedicated team and a component of a broader Forensic Mental Health Service.
Hours of operation	Business hours (depending on court hours)
Population profile	Individuals often have a complex presentation of severe mental illness, with high levels of comorbidity particularly substance use disorders, severe personality disorders, cognitive impairment, and who pose significant risk of harm to others.
Example service	Queensland Court Liaison Service

**3.** Community Forensic Mental Health Service

Service attribute	Details
Services provided	Aims to help individuals function effectively within the community and to manage the transition into and out of the community from institutional settings. Community Forensic Mental Health Services are delivered by multidisciplinary teams who either provide (a) direct case management/care coordination, assessment and treatment or (b) shared care/consultation, with primary responsibility remaining with a mainstream mental health service. Community Forensic Mental Health Services: ○Provide high quality mental health assessment, treatment and care to assist individuals with a significant risk of harm to others. ○*Assist prisoners with mental illness in transitioning back to the community and are important for facilitating appropriate referrals and follow up* ○*Assist in maintaining successful community living and preventing recurrence of problem behaviours.* ○*May be responsible for providing specialist psychological assessments to determine fitness and cognitive status.* Treatment may entail general mental health treatment or specialised interventions, targeted at problem behaviours such as sex offending, stalking or fire setting. *Treatment provided by Community Forensic Mental Health Services should include the continuing management of comorbid substance-use disorders where applicable.*
Key features	Community Forensic Mental Health Services are central to a mental health system’s capacity to provide continuity of care from community or mainstream mental health services, to institutional care (e.g. criminal justice service or a secure hospital setting) and back to community or mainstream mental health services. *It is important that services are delivered by experienced staff, such as forensic and trauma specialist clinicians, to minimise risks and assist during transition periods.* *Consumers are often on mandated bonds or parole, and as such, liaison with Correctional Services may be appropriate.*
Hours of operation	Extended hours (on-call service available)
Population profile	Individuals often have a complex presentation of severe mental illness that has been associated with serious offending, usually of a violent nature. Primary diagnoses often include psychotic disorders or severe affective disorders with high levels of comorbidity particularly substance use disorders, severe personality disorders or cognitive impairment, and who pose significant risk of harm to others.
Example service	N/A

**4.** Prison Mental Health Service

Service attribute	Details
Services provided	Aims to provide services to people affected by mental health problems who are incarcerated in an Australian prison. Core functions of the Prison Mental Health Service (PMHS) team include: ○Reception screening for the presence of mental illness; ○Early identification of mental illness in prisoners; ○High quality mental health assessment, treatment and care; and ○Transfer of consumers to hospital if necessary. *Prison Mental Health Services provide support to prison officers in the management of individuals with mental illness.* *Prison Mental Health Services are also involved in the identification, assessment, management and review of self-harm concerns and suicide prevention within the prison.* PMHS facilitate access to mental health services on release from custody and prepare individuals with complex mental health needs to integrate back into community. Prisoners with mental health issues may require assistance to access finances, accommodation, support networks and access to services. PMHS promote reintegration. *Prison Mental Health Services run periodic clinics in rural prisons. Telepsychiatry consultations are also provided to rural prisons as required.* *Prison Mental Health Services may be responsible for the delivery of clinical services to mental health units in prisons where applicable.*
Key features	PMHS are delivered by multidisciplinary teams which provide in-reach service to people affected by mental health problems and facilitate access to mental health care in prison, in dedicated prison mental health units and in general prison environments. PMHS will generally be an element of a Forensic Mental Health Service, with the capacity to provide continuity of care from community or mainstream mental health services, usually via the criminal justice system, to prison or a secure hospital setting, and return to the care of community or mainstream mental health services. *Prison Mental Health Services act as a point of contact for prison staff who are not trained in mental health. They provide training to equip prison staff with basic mental health responses and how to manage associated behaviours.*
Hours of operation	*Extended hours,* on-call service available
Population profile	Adults with severe and/or persistent mental illness or situational distress that has a significant impact on their functioning. Primary diagnoses often include psychotic disorders or severe affective disorders with high levels of comorbidity particularly substance use disorders, severe personality disorders or cognitive impairment. Prisoners with mild and moderate mental illness are considered to be the responsibility of primary care. Note: PMHS can be accessed by any person detained in a prison or detention centre. However, the predominant population is male and aged between 18 and 64. Indigenous Australians are over-represented and make up approximately 30% of Australian prisoners. Women are underrepresented, making up less than 9% of Australian prisoners, however the prevalence of mental disorder in female prisoners, particularly moderate and severe disorders, is approximately 1.5 higher than in male prisoners.
Example service	N/A

**5.** Acute Forensic Assessment Unit

Service attribute	Details
Services provided	Aims to provide short to medium-term inpatient assessment and treatment planning services for people in custody experiencing or suspected to be experiencing severe episodes of mental illness complicated by alleged or proven offending and who require further assessment in a secure setting. The core business is to provide multidisciplinary specialised assessment, evidence based, collaborative treatment planning, and initial interventions in a safe, therapeutic environment. Programs primarily provide specialist psychiatric assessment and treatment planning for people with acute episodes of mental illness, which are characterised by recent onset of severe clinical symptoms of mental illness associated with offending behaviour that led to a custodial outcome. The Acute Forensic Assessment Unit: ○*Provides trauma-informed practice and services performed are culturally secure and are carried out in close liaison with Indigenous workforce and/or specialist advisors.* ○*Provides timely access to specific care and treatment to meet consumers’ particular forensic and mental health needs.* ○*Provides an environment to address consumers’ diagnostic and offence-related issues.* ○*Provides social services for consumers, such as linkages with legal services, assistance with finding housing, and developing connections with community support systems.* *Provides physical health support, such as in monitoring and addressing antipsychotic side effects.*
Key features	*The Acute Forensic Assessment Unit may also accommodate high-risk civil patients (due to the severity of their risk toward themselves or others) or forensic patients who are not in custody.* Acute Forensic Assessment Units (bed based services) are an element of a forensic mental health service. The key characteristic of an Acute Forensic Assessment Service is ensuring assessment and treatment planning is provided in a setting which balances patient welfare and community safety. The clinical focus is on decreasing acuity to a level that can be treated in another environment such as ambulatory prison services, acute or sub acute forensic bed based services or mainstream mental health services. Gazetted ALOS 42 days
Hours of operation	24 hours/7 days
Population profile	Adults in custody with a possible or diagnosed severe mental illness accompanied by alleged or proven offending, which could not be adequately assessed, investigated or treated in prison or in a mainstream mental health service. Individuals often have a complex presentation of severe mental illness, with high levels of comorbidity particularly substance use disorders, severe personality disorders, cognitive impairment, and who pose significant risk of harm to others.

**6.** Acute Forensic Management Unit

Service attribute	Details
Services provided	Aims to provide short to medium-term inpatient assessment and treatment services for people experiencing or suspected to be experiencing severe episodes of mental illness associated with significant violent or other serious offending behaviour. The core business is to provide multidisciplinary specialised assessment, best practice, evidence based and collaborative planning, and interventions, in a safe, therapeutic environment. Programs primarily provide specialist psychiatric care for people with acute episodes of mental illness. These episodes are characterised by recent onset of severe clinical symptoms of mental illness associated with offending behaviour of a serious nature generally involving violence to others. The Acute Forensic Management Unit: ○*Provides trauma-informed practice and services performed are culturally secure and are carried out in close liaison with Indigenous workforce and/or specialist advisors.* ○*Provides recovery-focussed treatment and care.* Referrals may be received from the criminal justice system and/or mainstream mental health services.
Key features	Acute Forensic Management Units are a component of the continuum of stepped therapeutic care located in high security forensic hospitals. Individuals accessing this service pose a significant risk of harm to others and who require a higher level of physical, relational and procedural security than can be provided in mainstream mental health services. The key characteristic of Acute Forensic Management Units is ensuring treatment is provided in a setting which balances patient welfare and community safety while focusing on decreasing acuity to a level that can be treated in less intensive environments. Gazetted ALOS 56 days (needs benchmarking)
Hours of operation	24 hours/7 days
Population profile	Individuals often have a complex presentation of severe mental illness that has been associated with serious offending, usually of a violent nature. Primary diagnoses often include psychotic disorders or severe affective disorders with high levels of comorbidity particularly substance use disorders, severe personality disorders, cognitive impairment, and who pose significant risk of harm to others.
Example service	Thomas Embling Hospital (Victoria) The Frankland Centre (Western Australia) James Nash House (South Australia)

**7.** Sub Acute Forensic Unit

Service attribute	Details
Services provided	Aims to provide medium to long term treatment and rehabilitation in a safe, structured environment for individuals experiencing or suspected to be experiencing severe episodes of mental illness associated with significant violent or other serious offending behaviour. The Sub Acute Forensic Unit: ○Provides specialist behavioural and symptom management programs. ○Provides individualised and group rehabilitation programs aimed at maximising individual functioning and minimising the likelihood of repeat offending related to recurrent mental disorder. ○Supports the transition to safe management and monitoring in a less secure setting. ○Provides trauma-informed practice and services performed are culturally secure and are carried out in close liaison with Indigenous workforce and/or specialist advisors. ○Provides family-inclusive practice. Programs have a strong focus on safety, security and risk assessment and management but have the capacity to build and test an individual’s ability to manage their treatment needs as well as relationships, occupational capacity and activities of daily living within the safety of a secure perimeter.
Key features	Sub-acute Forensic Units are a component of the continuum of stepped therapeutic care are located in high security forensic hospitals. These units focus on addressing dynamic risk over a sustained period of time. Individuals accessing this service pose a significant risk of harm to others and require a higher level of physical, relational and procedural security than can be provided in mainstream mental health services. *Consumers within a Sub Acute Forensic Unit would typically access community rehabilitation programs.* Gazetted ALOS would typically be at least 12 months.
Hours of operation	24 hours/7 days
Population profile	Individuals often have a complex presentation of severe mental illness that has been associated with serious offending, usually of a violent nature. Primary diagnoses often include psychotic disorders or severe affective disorders with high levels of comorbidity particularly substance use disorders, severe personality disorders or cognitive impairment, and who pose significant risk of harm to others.
Example service	James Nash House (SA)

**8.** Non Acute Forensic Unit

Service attribute	Details
Services provided	Aims to provide long-term treatment and rehabilitation in a safe, structured environment for individuals with severe mental illness associated with significant violent or other serious offending behaviour. The Non Acute Forensic Unit: ○Provides specialist behavioural and symptom management programs. ○Provides Individualised and group rehabilitation programs aimed at maximising individual functioning and minimising the likelihood of repeat offending related to recurrent mental disorder. ○Supports the transition to safe management and monitoring in a less secure setting. ○*Provides trauma-informed practice and services performed are culturally secure and are carried out in close liaison with Indigenous workforce and/or specialist advisors.* Programs have a strong focus on safety, security and risk assessment and management but have the capacity to build and test an individual’s ability to manage their treatment needs as well as substance use risks, relationships, occupational capacity and activities of daily living within a secure perimeter. *An important function of Non Acute Forensic Units is providing psychologically informed practice for comorbid personality disorders.* *Residents of Non Acute Forensic Units attend peer groups and may attend substance abuse rehabilitation groups.*
Key features	Non-acute Forensic Units are a component of the continuum of stepped therapeutic care located in high security forensic hospitals. Individuals accessing this service pose a significant risk of harm to others and require a higher level of physical, relational and procedural security than can be provided in mainstream mental health services. Individuals may access the community for specific rehabilitation purposes, which is generally of limited duration with varying degrees of supervision in place. *Non Acute Forensic Units can also provide medium and low secure treatment settings.* While clinical need should determine length of stay in this unit, legal circumstances might also impact length of stay depending on the jurisdiction. Gazetted
Hours of operation	24 hours/7 days
Population profile	Individuals often have a complex presentation of severe mental illness that has been associated with serious offending, usually of a violent nature. Primary diagnoses often include psychotic disorders or severe affective disorders with high levels of comorbidity particularly substance use disorders, severe personality disorders or cognitive impairment, and who pose significant risk of harm to others.
Example service	Forensic Non Acute Inpatient Service (Rehab Program)(Western Australia)

**9.** Non Acute Forensic Community Rehabilitation Unit

Service attribute	Details
Services provided	Aims to provide long-term treatment and rehabilitation in a structured supportive environment for severe mental illness associated with significant violent or other serious offending behaviour. Programs have a strong focus on safety, security and risk assessment and management but have the capacity to build and test an individual’s ability to manage their treatment needs as well as relationships, occupational capacity and activities of daily living beyond a secure perimeter. Services include: ○Specialist behavioural and symptom management programs; ○Individualised and group rehabilitation programs aimed at maximising functioning and minimising the likelihood of repeat offending related to recurrent mental disorder; and Supporting the transition to safe management and monitoring in less secure settings.
Key features	Non-acute Forensic Community Rehabilitation Units are a component of the continuum of stepped therapeutic care for those who require extended rehabilitation, are located in a setting that facilitates access to clinical support services and should be linked with local mainstream mental health services. Individuals accessing this service pose a significant risk of harm to others and require a higher level of physical, relational and procedural security than can be provided in mainstream mental health services. These units are stand-alone, may be self-contained and are located in a low secure environment. Individuals may access the community for specific rehabilitation purposes. The expected outcomes are to maximise consumer welfare and reduce the potential for relapse and reoffending. *Support provided by Non-Government Organisations (NGOs) is a key aspect of this service.* Gazetted ALOS 12+ months
Hours of operation	24 hours/7 days
Population profile	Individuals often have a complex presentation of severe mental illness that has been associated with serious offending, usually of a violent nature. Primary diagnoses often include psychotic disorders or severe affective disorders with high levels of comorbidity particularly substance use disorders, severe personality disorders or cognitive impairment, and who pose significant risk of harm to others.
Example service	Ashton House (South Australia)
